# ACTH and Polymorphisms at Steroidogenic Loci as Determinants of Aldosterone Secretion and Blood Pressure

**DOI:** 10.3390/ijms18030579

**Published:** 2017-03-07

**Authors:** Scott M. MacKenzie, E. Marie Freel, John M. Connell, Robert Fraser, Eleanor Davies

**Affiliations:** 1 Institute of Cardiovascular and Medical Sciences, College of Medical, Veterinary and Life Sciences, University of Glasgow, Glasgow G12 8TA, UK; marie.freel@glasgow.ac.uk (E.M.F.); robertfraser36@gmail.com (R.F.); eleanor.davies@glasgow.ac.uk (E.D.); 2 Ninewells Hospital and Medical School, University of Dundee, Dundee DD1 9SY, UK; j.m.connell@dundee.ac.uk

**Keywords:** hypertension, ACTH, aldosterone, *CYP11B2*, *CYP11B1*, *CYP17A1*

## Abstract

The majority of genes contributing to the heritable component of blood pressure remain unidentified, but there is substantial evidence to suggest that common polymorphisms at loci involved in the biosynthesis of the corticosteroids aldosterone and cortisol are important. This view is supported by data from genome-wide association studies that consistently link the *CYP17A1* locus to blood pressure. In this review article, we describe common polymorphisms at three steroidogenic loci (*CYP11B2, CYP11B1* and *CYP17A1*) that alter gene transcription efficiency and levels of key steroids, including aldosterone. However, the mechanism by which this occurs remains unclear. While the renin angiotensin system is rightly regarded as the major driver of aldosterone secretion, there is increasing evidence that the contribution of corticotropin (ACTH) is also significant. In light of this, we propose that the differential response of variant *CYP11B2, CYP11B1* and *CYP17A1* genes to ACTH is an important determinant of blood pressure, tending to predispose individuals with an unfavourable genotype to hypertension.

## 1. Introduction

The importance of hypertension as a risk factor for cardiovascular and cerebrovascular disease is well established. In addition to the environmental and lifestyle factors known to influence an individual’s blood pressure, there is also a substantial heritable component, estimated to account for ~40% of the phenotype [1]. Over the last few decades, several large-scale studies have been mounted to identify the locations of the genetic polymorphisms underlying this effect but, so far, only 50 or so loci have been significantly associated with blood pressure, accounting for <5% of the heritable component. A recent study found that most of the heritability influencing systolic blood pressure (SBP) and diastolic blood pressure (DBP) is attributable to common genetic polymorphisms, defined as those with a minor allele frequency (MAF) greater than 0.1, located in non-coding regions that are probably *cis*-regulatory [2]. Identification of these unknown polymorphisms is highly desirable, as it will lead to greater understanding of the mechanisms underlying blood pressure homeostasis, thereby improving the treatment and differential diagnosis of hypertension. Investigators have employed contrasting strategies in this search, some favouring the hypothesis-free approach of the genome-wide association study (GWAS) on the basis that this may identify genes previously unsuspected genes of being significant mediators of blood pressure homeostasis. Others, ourselves included, have favoured a candidate gene approach, reasoning that many of the responsible loci are likely to be found among those participating in the blood pressure regulatory pathways. One such pathway is the biosynthesis of aldosterone, primarily under the regulation of the renin–angiotensin system.

Aldosterone is formed through sequential modifications to cholesterol catalysed by a series of steroidogenic enzymes present in the adrenocortical zona glomerulosa; the adjacent zona fasciculata, owing to a slightly different combination of enzymes, synthesises the major glucocorticoid cortisol [3]. There are several reasons to believe that the genes encoding these steroidogenic enzymes contribute to blood pressure variability. Firstly, aldosterone is a key regulator of blood pressure, and the secretion rates and plasma levels of aldosterone are heritable traits [4,5]. Secondly, severe disruption of this pathway by rare monogenic disorders affecting steroidogenic enzyme function and therefore steroid phenotype can dramatically affect blood pressure [6]. Given that these rare mutations have such severe outcomes, much of our work has been based on the hypothesis that more common but mild functional polymorphisms also exist at these steroidogenic loci. Such polymorphisms, influencing the expression or function of the steroidogenic enzymes, would have more subtle effects on steroid phenotype than their rarer, more severe counterparts but would nonetheless result in small but clinically significant increases in average blood pressure, ultimately predisposing carriers in combination with other heritable and environmental factors to hypertension.

In this article, we summarise our research into common functional polymorphisms, at three steroidogenic loci that we believe contribute to the heritable component of blood pressure: *CYP11B2*, *CYP11B1* and *CYP17A1*. We then discuss the importance of corticotropin (ACTH) to the regulation of all three genes, present evidence to suggest that this is an underappreciated factor in the long-term regulation of aldosterone secretion, and propose that the differential response of these polymorphic loci to ACTH may predispose their carriers to raised blood pressure and, ultimately, hypertension.

## 2. *CYP11B2*

Given its functional role, the *CYP11B2* gene is an obvious blood pressure locus candidate. It encodes aldosterone synthase (responsible for the three final stages of aldosterone biosynthesis, from 11-deoxycorticosterone onward), which is the only adrenocortical steroidogenic enzyme unique to the production of aldosterone. The renin-angiotensin system (RAS) and raised extracellular potassium concentration are the principal stimulators of *CYP11B2* expression; ACTH is generally regarded as having only a short-lived effect, of far less physiological significance than the other factors [3]. The potential for modified *CYP11B2* gene expression to cause significant blood pressure effects is most starkly illustrated by the rare monogenic condition, glucocorticoid-remediable aldosteronism (GRA), where unequal crossing-over of this locus with that of the *CYP11B1* gene (see below), leads to vastly increased expression of aldosterone synthase, elevating levels of aldosterone and resulting in severe hypertension [6]. Clearly, any *CYP11B2* polymorphism with a role in population blood pressure variation should be much more common than GRA-causing mutations, and less pronounced in its effect. In order to support the hypothesis that *CYP11B2* is an important blood pressure locus, it was therefore necessary to demonstrate firstly that such common polymorphisms exist at the *CYP11B2* locus and secondly that they have a functional effect on steroid phenotype. In the 30 years since its sequence was first published [7], numerous common *CYP11B2* polymorphisms have indeed been identified, the most studied of which is the rs1799998 C/T single-nucleotide polymorphism (SNP), also referred to as “‑344” given its base position relative to the gene transcription start site. It is extremely common—its minor allele frequency (MAF) is ~0.43 in Western European populations [8] and studies have shown that its T allele associates with higher excretion rate of the major urinary aldosterone metabolite, tetrahydroaldosterone (THaldo) [9]. However, no clear consensus has emerged on whether this polymorphism associates with changes in blood pressure [10,11,12,13]. Meta-analysis of 42 individual studies did show an association of rs1799998 with essential hypertension, but not with systolic or diastolic blood pressure [10]. According to in vitro studies, the effect of this SNP on *CYP11B2* gene transcription is significant only following stimulation with angiotensin II; even then, the effect is small [14]. We believe this lack of clarity regarding rs1799998 effect may be due to its having no functional impact itself, and from it being in tight but not complete linkage disequilibrium (LD) with a functional polymorphism lying nearby. One such polymorphism, which we have studied extensively, is rs13268025.

The rs13268025 polymorphism is a C/T SNP positioned 1651 bases upstream of the *CYP11B2* transcription start site; its alleles are of almost equal frequency in our West of Scotland study population (MAF = 0.47) and it displays a high degree of LD with rs1799998 (D’ > 0.95). Our study of 60 normotensive subjects showed that those homozygous for the C allele excreted significantly higher quantities of THaldo in a 24 h period than the T homozygotes [15]. Reporter gene assays compared the transcriptional activity of the *CYP11B2* promoter in its alternative rs13268025 forms and were consistent with our in vitro studies: this SNP at the 1651 position significantly affected gene activity, with the C form being more transcriptionally active under basal conditions. This difference in activity between the alternative alleles became even more pronounced following angiotensin II stimulation. We have linked this change in activity to the disruption of a binding site for the transcriptional repressor molecule apurinic/apyrimidinic endodeoxyribonuclease 1 (APEX1) at the 1651 position. APEX1 has lower affinity for the C form of rs13268025, reducing its inhibitory effect on *CYP11B2* transcription [15]. (Interestingly, although knockout of APEX1 is lethal in mice, heterozygous null mice are viable and have markedly elevated blood pressure [16].)

So, in rs13268025 we have a common polymorphism, carried by approximately half of our study population in either homozygotic or heterozygotic form, which alters transcriptional activity of the aldosterone synthase gene and associates with a corresponding change in steroid phenotype, while also being physically linked to a well-researched polymorphism at the same locus (rs1799998), which has itself been significantly associated with functional effects. This identifies *CYP11B2* as a locus harbouring at least one common polymorphism of functional effect that could influence blood pressure and predispose carriers of the “derepressed” C allele to hypertension. 

Surprisingly, the strongest association of *CYP11B2* genotype with steroid phenotype to emerge from these studies was not with aldosterone but with 11-deoxysteroids, which one would expect to be primarily influenced by *CYP11B1*, the gene encoding the 11β-hydroxylase enzyme, rather than *CYP11B2* [17]. This relationship between *CYP11B2* polymorphisms and indices of *CYP11B1* function is not as unlikely as it might first appear; the two genes lie side by side on human chromosome 8, approximately 40 kilobases apart, and our studies show that their physical linkage could underlie this observation.

## 3. *CYP11B1*

*CYP11B1* encodes 11β-hydroxylase responsible for the final catalytic conversion in the biosynthesis of cortisol. The gene is expressed within the adrenocortical zona fasciculata, and to a lesser extent the zona reticularis, predominantly under the regulation of ACTH, acting through cyclic AMP (cAMP) as a second messenger. The strict zonal distribution of expression within the normal adrenal gland dictates that *CYP11B1* is not coexpressed with *CYP11B2*, expression of which is confined to the zona glomerulosa [6]. Nevertheless, strong association of the *CYP11B2* polymorphism rs1799998 with indices of 11β-hydroxylase activity, such as levels of urinary 11-deoxycortisol metabolites, have been observed in several studies [17,18,19]. This is the result of LD extending across the entire digenic locus, causing certain *CYP11B2* polymorphisms to associate with functional *CYP11B1* SNPs and, therefore, 11β-hydroxylation of steroids.

One such functional SNP at *CYP11B1* is the G/T SNP rs142570922, located 1889 bases upstream of the transcription start site. Analysis of 408 hypertensive subjects show that, in comparison to carriers of the major G allele (where MAF = 0.49), those with the minor T allele are predisposed to a higher ratio of tetrahydrodeoxycortisol (THS) to cortisol metabolites in their urine, reflecting lower 11β-hydroxylation efficiency [20]. Subsequent analysis of adrenal tissue from individuals heterozygous for rs142570922 shows that this T form of *CYP11B1* is transcribed at a significantly lower rate than its G form; lower expression is consistent with the observed inefficiency of 11β-hydroxylation [8].

Given the presence of a common functional SNP within *CYP11B2* that is significantly associated with levels of cortisol, a steroid that influences blood pressure, there is strong evidence to suggest that *CYP11B1*, like *CYP11B2*, is a blood pressure locus. As with *CYP11B2*, support comes from rare monogenic disorders of *CYP11B1*. Various inactivating mutations at this locus have been described that result in deficient 11β-hydroxylation and, depending on their severity, reduced or completely absent cortisol production. The consequent accumulation of ACTH-dependent 11-deoxycorticosterone (DOC), which has mineralocorticoid properties, can in such instances be high enough to result in severe hypertension. Efforts to attain normal levels of cortisol lead to elevated ACTH secretion and hyperplasia of the adrenal cortex [21].

The observation that these two key genes of the corticosteroid pathway are each capable independently of influencing blood pressure would be interesting enough in itself, but their physical linkage adds an extra dimension that is likely to augment their hypertensive role. As mentioned previously, our analysis of the digenic *CYP11B1*/*CYP11B2* locus on chromosome 8 shows that LD extends across both genes, such that the presence of a particular allele at a polymorphic site in *CYP11B1* is a strong indicator that a particular polymorphic form exists within *CYP11B2.* This is the case for the two functional SNPs we have described: the C form of rs13268025 at *CYP11B2*, which is associated with a raised aldosterone excretion rate, is in significant LD with the T form of rs142570922 at *CYP11B1*, which is associated with reduced 11β-hydroxylation. These two potentially hypertensive alleles tend, therefore, to be co-inherited. Furthermore, we propose that their combined effect on blood pressure may be synergistic.

Our proposed mechanism for this arises from three separate elements. The first two have already been mentioned with reference to severe monogenic 11β-hydroxylase deficiency: the massive rise in ACTH secretion and the expansion of adrenocortical volume it causes in an attempt to restore cortisol biosynthetic capacity. The third element concerns a known but, we would argue, underappreciated factor in aldosterone biosynthesis: its regulation by ACTH. ACTH acutely stimulates aldosterone biosynthesis by both increasing adrenal blood flow and interacting with specific G protein-coupled receptors in the zona glomerulosa, which influences the expression of steroidogenic genes containing cAMP-responsive elements; these include *CYP11B2* [22]. However, several studies showed that prolonged administration of ACTH could not sustain aldosterone secretion and that it would fall back to basal levels after approximately three to five days [23,24,25]. Although the mechanism for this suppression remains unknown, such findings feed the general perception that ACTH cannot influence aldosterone biosynthesis for anything other than short periods of time, and that its long-term effect is inhibitory. However, we have proposed that the suppression of aldosterone biosynthesis under chronic exposure to ACTH is a consequence of abnormally high doses, whereas chronic exposure to only mildly increased endogenous ACTH secretion could permit the sustainable stimulation of aldosterone secretion [3]. We examine evidence for this in greater detail below but, for now, let us consider the consequences for individuals carrying the T form of rs142570922 at *CYP11B1* if such a prolonged and increased ACTH influence on aldosterone were to exist. As the indisputably prime regulator of cortisol biosynthesis and of *CYP11B1* expression, carriers of this polymorphism would experience mild, relative 11β-hydroxylase inefficiency due to the slight reduction in transcriptional efficiency it causes. Such individuals would have to prevent the small but significant drop in cortisol biosynthesis that would result by increasing ACTH secretion slightly but chronically. Although analogous to the increased ACTH release observed in severe 11β-hydroxylase deficiency, we argue that this milder form would differ in two key ways. Firstly, hypertrophy of the adrenal cortex would be a much slower and more gradual process, reflecting the relatively small rise in ACTH level. Secondly, the magnitude of this increase would not be sufficient to trigger the suppression of aldosterone secretion commonly observed when the adrenal cortex is chronically exposed to higher levels of ACTH. Instead, over the lifetime of its carriers, the rs142570922 T polymorphism at *CYP11B1* would induce very gradual hypertrophy and a mild but sustained elevation of aldosterone biosynthesis through ACTH stimulation. As we have observed, this polymorphism cannot be considered in isolation: it is highly likely to be co-inherited alongside the C form of rs13268025 at *CYP11B2*, which itself also predisposes to slightly increased aldosterone secretion. Furthermore, as a component of the adrenocortical hypertrophy consequent upon raised ACTH, there is likely to be zona glomerulosa hypertrophy, further increasing its aldosterone biosynthetic capacity and/or its responsiveness to stimulators of aldosterone secretion such as angiotensin II (or ACTH itself). In summary, due to the interplay of these two linked polymorphisms at adjacent steroidogenic genes, we anticipate a small but chronic rise in ACTH which, over a lifetime, significantly increases aldosterone secretion and drives genetically predisposed carriers toward hypertension.

This mechanism was first proposed several years ago [26] and, although we have since been able to refine and confirm certain details, certain aspects remain unproven. This is largely due to the subtle and extremely long-term nature of the proposed effects, which are too small to measure or detect, and to the difficulty in demonstrating that such a small increase in ACTH secretion would stimulate aldosterone secretion without causing the inhibition of secretion many still associate with long-term exposure. There is some further supporting evidence: in a small group of patients subjected to low-dose dexamethasone suppression, those who were homozygous for the T form of rs142570922 at *CYP11B1* had a small but significantly higher level of plasma ACTH compared to G homozygotes. Plasma cortisol levels did not change significantly between the different genotypes, but there was an apparent trend towards a raised cortisol:ACTH ratio in the T group [27]. This supports the existence of an increased ACTH drive in these individuals sustaining cortisol levels, in line with our proposal. Further investigation is ongoing to verify this hypothesis. For example, we have recent preliminary data from 100 normotensive volunteers who were infused with corticotropin-releasing hormone; this suggests that individuals with the genotype rs142570922 T at *CYP11B1* and/or rs13268025 G at *CYP11B2* respond more vigorously in terms of ACTH and ACTH-dependent steroids (cortisol, corticosterone and deoxycortisol) than individuals of the alternative genotypes [28].

Meanwhile, data emerging from GWAS specifically designed to identify loci of importance in blood pressure regulation also drew attention to the corticosteroid pathway, but the locus they were consistently highlighting was not *CYP11B2* or *CYP11B1,* but *CYP17A1* [29,30].

## 4. *CYP17A1*

Over the last decade, GWAS methodology has been applied to numerous complex traits, including blood pressure, with the intention of discovering associations between disease and common genetic polymorphisms. Its potential to identify loci previously unsuspected to influence certain phenotypes was presented as a key advantage over the candidate gene approach. However, as GWAS results emerged, there was a common perception that such studies were not delivering on their initial promise and that a great number of relevant loci remained undetected (false negatives). Counter-arguments hold that GWAS was never intended to identify all loci contributing to a particular trait, and that such studies have led to new and meaningful new biological knowledge, while also confirming the importance of key genes and pathways previously suspected of playing a role in disease [31].

Data from blood pressure GWAS conducted on European subjects by the Global BPgen and CHARGE consortia were published in 2009 [29,30]. These, combined with further meta-analysis [32], identified a total of 28 loci significantly associated with systolic and/or diastolic blood pressure. *CYP11B1* and *CYP11B2* were not among them. However, a region on the long arm of chromosome 10 at or near the *CYP17A1* gene was consistently identified, and was associated with the greatest effect size of all the 28 loci: ~1 mm Hg of systolic blood pressure per allele. The tag marker employed by Global BPgen at this locus was rs11191548, which lies within a cluster of six genes, including *CYP17A1*; the tag marker used by CHARGE was rs1004467 [30], found within intron 3 of *CYP17A1* [29]. This association is not limited to European populations [33,34,35,36,37,38]. The same locus has recently been associated with birthweight, which itself inversely correlates to blood pressure in later life [39]; there is also evidence that control of aldosterone secretion associates with birthweight and that this effect may derive from the hypothalamic-pituitary-adrenal (HPA) axis [40]. In light of the compelling GWAS data and the known role of this gene in corticosteroid biosynthesis and blood pressure, we investigated it further.

The *CYP17A1* gene encodes a microsomal cytochrome P450 enzyme commonly named for its catalytic function: 17α-hydroxylase. In the adrenal zona fasciculata, this enables the enzyme to convert pregenenolone to 17α-hydroxypregnenolone and progesterone to 17α-hydroxyprogesterone, which is essential to the synthesis of cortisol. However, it also possesses a lyase function enabling the cleavage of the C17,20 bond of each of these products. In the zona reticularis, this permits the production of dehydroepiandrosterone and androstenedione (*CYP17A1* is also expressed in the gonads) [6,41]. Here, we will focus on its 17α-hydroxylase activity in the zona reticularis.

Like *CYP11B1*, the *CYP17A1* gene is primarily regulated by ACTH; this activates signalling cascades involving the intracellular second messenger cAMP, which promotes gene expression through the binding of transcription factors including steroidogenic factor 1 (SF-1), GATA-binding factor 6 (GATA-6) and sterol regulatory element-binding protein 1 (SREBP-1) [42]. Loss of 17α-hydroxylase activity due to *CYP17A1* mutation can, as with 11β-hydroxylase deficiency, reduce cortisol synthesis and lead to a surge in ACTH secretion, plus the accumulation of DOC [6]. This results in hypertension, DOC-dependent sodium accumulation, the suppression of aldosterone secretion and hypokalaemia; grossly raised levels of corticosterone compensate for the lack of cortisol. Sexual development is also affected, reflecting the role of *CYP17A1* in androgen production. We therefore hypothesised that, as with *CYP11B1* and *CYP11B2*, functional polymorphisms exist at the *CYP17A1* locus, which alter the corticosteroid phenotype and influence blood pressure. To explain the blood pressure GWAS associations, any such polymorphism at *CYP17A1* would also have to be in LD with the tag SNPs employed by those studies and have a fairly common frequency within the general population.

Our initial screen of the entire *CYP17A1* locus in a cohort of 60 normotensive volunteers identified 36 polymorphisms, of which 24 were common, with a MAF > 0.05 [43]. Of these 24, none were missense and several were intronic, although not at splice sites. We therefore focused our attention on the 7 SNPs that lay between the transcription start site and the limit of our sequencing, ~2 kilobases upstream, reasoning that these might influence transcriptional regulation. Linkage analysis identified two distinct LD blocks: while one block contains six of the SNPs, but none of the GWAS tag SNPs, the other contains the remaining *CYP17A1* SNP rs138009835 and is in significant LD with the rs1004467 SNP, shown by the CHARGE GWAS to associate with blood pressure. Therefore, of all the polymorphisms identified during our screen of the *CYP17A1* locus, only rs138009835, a G/A SNP with a MAF of 0.11, located 1.8 kilobases upstream of the transcription start site, emerges as a SNP plausibly underlying the blood pressure GWAS association.

Our subsequent analysis of the SNP rs138009835 in 232 hypertensive members of the British Genetics of Hypertension (BRIGHT) cohort showed that individuals homozygous for the major G allele had significantly increased THaldo excretion rates but no association with the excretion rates of metabolites of cortisol, deoxycortisol or androgens [43]. Reporter gene analysis also showed this G allele to associate with significantly higher transcriptional activity in vitro, under basal conditions and following dibutyryl cAMP stimulation (which is used to imitate ACTH in the H295R adrenocortical cell model that lacks ACTH receptors). In summary, the G form of rs138009835 associates with higher *CYP17A1* gene expression in vitro, increased aldosterone secretion in vivo and, through its linkage to the A allele of the rs1004467 tag SNP, raised blood pressure.

Superficially, this appears to present a simple mechanism whereby functional changes in the steroidogenic *CYP17A1* gene might affect aldosterone phenotype, with a corresponding effect on blood pressure. However, it is not immediately clear how such changes in *CYP17A1* function translate into altered aldosterone production, given that *CYP17A1* is not expressed in the zona glomerulosa and therefore has no direct role in aldosterone biosynthesis. One possibility is that *CYP17A1* and aldosterone share a common regulatory factor that connects the two and underlies our observed effect. Interestingly, previous clinical studies conducted by our group show highly significant correlation of urinary THaldo excretion with that of cortisol metabolites [44]. Data from that same study also show correlation of these with urinary androgen metabolite excretion, implying that aldosterone, cortisol and androgens are under the regulation of a common factor. We propose that this common factor is ACTH, and that it is a significant regulator of these steroids throughout life.

## 5. *ACTH*

Due to the presence of ACTH/cAMP-responsive elements at each gene promoter, *CYP11B1*, *CYP11B2* and *CYP17A1* transcription are all regulated by ACTH [22]. While the role of ACTH in regulating *CYP11B1* and *CYP17A1* (and by extension cortisol and androgen secretion) is relatively straightforward, its control of *CYP11B2* and aldosterone production in the zona glomerulosa is more complex and not fully understood [45]. Our proposal that common polymorphisms of these three genes have a significant influence over aldosterone levels rests, in part, on ACTH having a more significant and enduring role in regulating its secretion than has generally been acknowledged. Evidence exists to support this view.

Although early studies suggested that chronic ACTH might ultimately inhibit aldosterone secretion, this is not necessarily the case. Continuous ACTH infusion does indeed cause aldosterone to rise then fall back to baseline levels within 72 h, but ACTH administered in a pulsatile manner that more closely models its actual secretion maintains aldosterone secretion at a raised level over this time period [46]. Furthermore, ACTH appears to be an extremely potent regulator of aldosterone, capable of eliciting its secretion at doses far lower than those required for cortisol or DHEA [47]. That being the case, could ACTH be a significant driver of aldosterone secretion? Correlation does exist between ACTH and aldosterone levels; each follows a circadian rhythm, peaking in the morning and falling throughout the day [48]. While this synchronization does suggest a causative relationship between the two, elements of the RAAS also display diurnal variation—albeit less consistently—and its contribution to this effect cannot be ruled out [49].

ACTH is also released following physical or psychological stress and a recent study by Markou et al. suggests not only that ACTH release driven by such stress can promote aldosterone secretion, but that certain individuals are highly sensitive to this stimulus. The investigators recruited 143 essentially hypertensive subjects, carefully phenotyped to exclude any cases of primary aldosteronism. To simulate low-grade chronic stress, this cohort was then given ultra-low doses (0.03 µg) of ACTH. This caused a rise in plasma cortisol levels indistinguishable between the hypertensives and a normotensive control group over the 30-min study period. However, analysis of plasma aldosterone levels revealed that the essential hypertensives actually consisted of two distinct subgroups: approximately three-quarters had a small but distinct rise in plasma aldosterone, again identical to that of the normotensives. The remaining quarter, though, were termed “hyper-responders” due to their aldosterone levels being approximately four times higher than those of the other subjects. The aldosterone:renin ratio of hyper-responders was also significantly elevated, suggesting the stimulatory effect of ACTH in these individuals occurs independently of the RAAS. Subjects then underwent a treadmill test, as a form of acute physical stress. Again, the hyper-responders produced inappropriately high levels of aldosterone when compared to the remainder of the hypertensives and the normotensive group, whose responses were indistinguishable from one another; in contrast, the cortisol responses did not differ significantly across hyper-responders, other hypertensives or normotensives. Consequently, the authors proposed that the glomerulosa cells of the hyper-secretors are “primed by stress-induced ACTH secretion, which makes them more sensitive to any increase in either ACTH or renin/angiotensin II levels” and that this could be related to “gene mutations increasing the zona glomerulosa responsiveness to excitatory stimuli” [50]. As John Funder concluded in his commentary on this study, “When validated, attention should focus on ACTH and on the mechanism(s) whereby it elicits an exaggerated aldosterone response in a substantial minority of patients otherwise classified as essential hypertensives” [51].

## 6. ACTH Interaction with Polymorphic Steroidogenic Loci as a Determinant of Aldosterone Secretion and Blood Pressure

ACTH levels directly influence cortisol and androgen levels, and vice versa. Rare monogenic deficiencies at *CYP11B1* and *CYP17A1* demonstrate the impact of these loci on circulating levels of ACTH through feedback mechanisms. However, as we have described, there is now compelling evidence that ACTH is a more significant driver of aldosterone secretion than previously thought. This implies the existence of a complex and dynamic situation whereby three different sets of components each interact with and modulate the levels of one another, namely:

(1) circulating levels of ACTH;
(2) gene expression of ACTH-responsive steroidogenic loci (including *CYP11B1* and *CYP17A1*, but also *CYP11B2*);
(3) circulating levels of corticosteroids (including cortisol and androgens, but also aldosterone).

A change in any one of these would alter the states of the other two (and probably of other, as yet unidentified) components involved in the homeostasis of this highly dynamic system.

Altered responsiveness of any of these components to one another would tend to push individuals towards higher or lower blood pressure, adding further complexity to this system. We propose that such changes in sensitivity result from common polymorphisms at *CYP11B1*, *CYP17A1* and *CYP11B2*, each of which are capable of significantly influencing these genes’ expression and thereby shifting the balance of circulating steroids. Therefore, an individual’s ability to secrete aldosterone in response to a given dose of ACTH would be significantly determined by the array of functional polymorphisms at key steroidogenic loci. This is analogous to the ACTH sensitivity observed by Markou et al. [50]. Their proposal that polymorphisms at the *KCNJ5* gene might contribute toward ‘hyper-responsiveness’ was not borne out by their sequencing of this locus. The genotyping of key SNPs at *CYP11B2*, *CYP11B1* and *CYP17A1* in such individuals would be of great interest.

We acknowledge that the observed effects of the three key polymorphisms we have described at *CYP11B1*, *CYP11B2* and *CYP17A1* are not entirely consistent with a predisposition to hypertension. Specifically, while we found that the form of *CYP17A1* causing higher activity is apparently associated with raised blood pressure, this runs contrary to the evidence that 17α-hydroxylation has an inverse relationship with blood pressure [52]. However, we propose that interaction between ACTH and these three loci is highly complex, and probably incorporates further factors in order to ultimately determine blood pressure. Therefore, we must be cautious in extrapolating the consequences of an isolated effect observed in vitro to the full complex system in vivo. The fact remains that genotype significantly affects function and will have consequences that must be integrated into the developing model.

## 7. Conclusions

In this article we have summarised evidence that common functional polymorphisms exist at the *CYP11B1*, *CYP11B2* and *CYP17A1* loci, and have hypothesised that each may predispose to hypertension. Previously, we had proposed ACTH via the *CYP11B1* and *CYP11B2* polymorphisms to be a key mediator in this hypertensive drive. Since then, data identifying the ACTH-regulated *CYP17A1* gene as a key blood pressure locus, the association of a common functional *CYP17A1* polymorphism with aldosterone phenotype and the discovery that a sizeable proportion of essential hypertensives secrete inappropriately high levels of aldosterone in response to ACTH has led us to develop and refine this initial model. Our future work will focus on establishing whether these polymorphisms contribute towards the hyper-secretion of aldosterone in response to ACTH.
